# A randomised controlled trial to determine the clinical and cost effectiveness of thulium laser transurethral vaporesection of the prostate (ThuVARP) versus transurethral resection of the prostate (TURP) in the National Health Service (NHS) – the UNBLOCS trial: a study protocol for a randomised controlled trial

**DOI:** 10.1186/s13063-017-1916-5

**Published:** 2017-04-17

**Authors:** Jo Worthington, Hilary Taylor, Paul Abrams, Sara T. Brookes, Nikki Cotterill, Sian M. Noble, Tobias Page, K. Satchi Swami, J. Athene Lane, Hashim Hashim

**Affiliations:** 1 Bristol Randomised Trials Collaboration (BRTC), School of Social and Community Medicine, University of Bristol, Canynge Hall, 39 Whatley Road, Bristol, BS8 2PS UK; 2 Bristol Urological Institute, Level 3, Learning and Research Building, North Bristol NHS Trust, Southmead Hospital, Bristol, BS10 5NB UK; 3 Department of Urology, The Newcastle upon Tyne Hospitals NHS Foundation Trust, Freeman Hospital, Freeman Road, High Heaton, Newcastle upon Tyne, NE7 7DN UK; 4 NHS Grampian, Department of Urology, Aberdeen Royal Infirmary, Foresterhill, Aberdeen, AB25 2ZN UK

**Keywords:** UNBLOCS, Prostate, Surgery, Lower urinary tract symptoms, Benign prostatic obstruction, Randomised controlled trial, Transurethral resection of the prostate, TURP, Thulium laser transurethral vaporesection of the prostate, ThuVARP

## Abstract

**Background:**

Transurethral resection of the prostate (TURP) has been the standard operation for benign prostatic obstruction (BPO) for 40 years, with approximately 25,000 procedures performed annually, and has remained largely unchanged. It is generally a successful operation, but has well-documented risks for the patient. Thulium laser transurethral vaporesection of the prostate (ThuVARP) vaporises and resects the prostate using a surgical technique similar to TURP. The small amount of study data currently available suggests that ThuVARP may have certain advantages over TURP, including reduced blood loss and shorter hospital stay, earlier return to normal activities, and shorter duration of catheterisation.

**Design:**

A multicentre, pragmatic, randomised, controlled, parallel-group trial of ThuVARP versus standard TURP in men with BPO. Four hundred and ten men suitable for prostate surgery were randomised to receive either ThuVARP or TURP at four university teaching hospitals, and three district general hospitals. The key aim of the trial is to determine whether ThuVARP is equivalent to TURP judged on both the patient-reported International Prostate Symptom Score (IPSS) and the maximum urine flow rate (Qmax) at 12 months post-surgery.

**Discussion:**

The general population has an increased life expectancy. As men get older their prostates enlarge, potentially causing BPO, which often requires surgery. Therefore, as the population ages, more prostate operations are needed to relieve obstruction. There is hence sustained interest in the condition and increasing need to find safer techniques than TURP. Various laser techniques have become available but none are widely used in the NHS because of lengthy training required for surgeons or inferior performance on clinical outcomes. Promising initial evidence from one RCT shows that ThuVARP has equivalent clinical effectiveness when compared to TURP, as well as other potential advantages. As ThuVARP uses a technique similar to that used in TURP, the learning curve is short, potentially making it also very quickly generalisable. This randomised study is designed to provide the high-quality evidence, in an NHS setting, with a range of patient-reported, clinical and cost-effectiveness outcomes, which will underpin and inform future NICE guidance.

**Trial registration:**

ISRCTN registry, ISRCTN00788389. Registered on 20 September 2013.

**Electronic supplementary material:**

The online version of this article (doi:10.1186/s13063-017-1916-5) contains supplementary material, which is available to authorized users.

## Background

The prostate gland sits at the exit of the bladder like a collar, and as men get older their prostates enlarge. This can commonly result in either urinary retention, an inability to completely empty the bladder, or in bothersome lower urinary tract symptoms (LUTS) secondary to benign prostatic obstruction (BPO), such as slow and intermittent urinary stream. These conditions severely affect a man’s quality of life resulting in worsening physical and social functioning, vitality, and mental health [1]. Bothersome LUTS secondary to BPO with an International Prostate Symptom Score (IPSS) of at least 11 and a maximum urine flow rate (Qmax) less than 15 ml/s affects 2.5 million men aged 40–79 in the UK, with 44,000 new cases diagnosed annually [2]. LUTS may be treated by watchful waiting or drugs, but many will require prostate surgery, including almost all men who present in urinary retention.

Around 25,000 prostate operations are performed annually in the UK for men with BPO to relieve obstruction. Transurethral resection of the prostate (TURP), the gold standard operation, accounts for around 80% of these operations. TURP has been used widely for the last 40 years, and although it is generally a successful procedure, it is associated with small but significant risks. It has a 30-day mortality of 0.3%, and a range of morbidities including transurethral resection (TUR) syndrome (1%), which is due to the absorption of irrigating fluid leading to confusion and collapse; haemorrhage during the operation (transfusion rate: 5%); and subsequent urinary tract infections (up to 20%) [3]. These morbidities result in delayed discharge and increased re-admissions, increased primary care resource utilisation, considerable distress to patients and additional costs to the National Health Service (NHS).

The well-known risks for both mortality and morbidity from TURP have meant that many alternatives have been assessed. Various laser alternatives have been marketed, but none have become widely used. This is due, in part, to a long learning curve, or inferior performance regarding clinical outcomes, reducing the wider uptake. In fact, Hospital Episode Statistics (HES) data shows that the percentage of laser procedures has only increased in England by 2% over the past 5 years, from 2894 in 2009/10 to 2958 in 2011/12. This is despite the accepted advantages of laser prostatectomy, including lower risk of peri-operative complications, shorter catheterisation time and reduced hospital stay [4]. Recent National Institute for Health and Care Excellence (NICE) clinical guidelines CG97 recommend offering TURP or holmium laser enucleation (HoLEP) for BPO surgery [5]. Although HoLEP is a long-established effective procedure, it is only used in a few centres, due to a very long learning curve reducing its generalisability, and indeed NICE recommends that this procedure is only performed in centres specialising in the technique.

This study, UriNary oBstruction relieved by Laser Or Conventional Surgery (UNBLOCS), is evaluating a new laser technique called thulium laser transurethral vaporesection of the prostate (ThuVARP). A thulium laser technique has been chosen which vaporises and resects the prostate because it uses a surgical technique similar to TURP, and will therefore enable a short learning curve making it quickly generalisable. It was first made available in 2004 in the UK but has only been compared in one randomised controlled trial (RCT) in China against TURP [6]. Based on this RCT and one non-randomised prospective controlled trial with small and medium-sized prostates [7], the European Association of Urology (EAU) guidelines have stated that ThuVARP showed equivalent efficacy in comparison with TURP [8]. However the thulium patients had shorter catheterisation and hospitalisation times, with adverse events being lower than for TURP (intra-operative and post-operative bleeding; level of evidence 1b).

NICE in its 2010 Male LUTS Guidelines [5] stated that the evidence base is inadequate to give clear guidance in terms of clinical and cost effectiveness of laser vaporesection techniques. NICE identified that research in this area, in the form of a randomised controlled trial, would help inform future guidance on the use of laser vaporesection techniques for men with LUTS or urinary retention, who need surgery.

### Rationale for the trial

The general population has an increased life expectancy, resulting in an ageing population. As BPO is a disease of older men, the number of patients with the condition is expected to grow by almost 50% by the year 2025, increasing the need for BPO surgery [2]. Furthermore, as the operation is increasingly conducted on older men (42% of the TURP operations in 2014–2015 were on patients older than 75 years), the risks of surgery associated with TURP will continue to increase. There is therefore sustained interest in the condition and increasing need to find safer techniques than TURP. The potential advantages of reduced blood loss, shorter hospital stay and earlier return to normal activities make laser vaporesection techniques attractive to both patients and health care providers. ThuVARP would allow urologists to operate on a wider range of men, including potentially those who are more frail and older, but with less risk. However, there is uncertainty about the degree of symptom improvement and improvement in quality of life in the short and longer term, which this trial addresses. Now is the ideal time to conduct the trial as the procedure has not yet been widely taken up across the UK.

An additional reason for early evaluation of ThuVARP is the promise it offers to convert BPO surgery from an inpatient operation into a day-case procedure. Shortened stay is increasingly important for the NHS both because of the increasing cost of inpatient beds, shortage of inpatient beds due to an ageing and increasingly co-morbid population, and the risk longer hospital stays present for such patients, for example of hospital-acquired infections.

In summary, although there is little existing work on ThuVARP, promising initial evidence from one RCT suggests that ThuVARP has equivalent clinical effectiveness when compared to TURP, albeit in a single Chinese centre. Our randomised study is designed to provide the high-quality evidence, in an NHS setting with a range of patient-reported, clinical and cost-effectiveness outcomes, which will underpin and inform future NICE guidance.

## Methods/trial design

### Study aims and objectives

The key aim of this research is to determine whether ThuVARP is equivalent to TURP in men with BPO treated within the NHS, judged on a patient-reported symptom severity score (IPSS) and the maximum urine flow rate (Qmax).

We will answer the following primary question: what is the relative clinical effectiveness of ThuVARP and TURP in improving patient-reported LUTS as measured by the International Prostate Symptom Score (IPSS) patient-reported questionnaire, and the objective measure of maximum urine flow rate (Qmax), 12 months after surgery?

Secondary research questions are:

1. How do the two procedures compare in terms of peri-operative outcomes?
2. What is the cost-effectiveness of ThuVARP as compared to TURP in terms of the two primary outcomes and quality-adjusted life years (QALYs: the primary economic outcome)?
3. What is the comparative impact of each treatment on patient-reported LUTS, erectile function, quality of life and general health?
4. What is the comparative satisfaction of men with each type of surgery?
5. What is the comparative effectiveness of these operations in men who present with LUTS as opposed to urinary retention?
6. What are men’s experiences of both procedures, including those presenting with LUTS or urinary retention?

### Trial design

This is a multicentre, pragmatic, randomised, controlled, parallel-group trial of thulium laser transurethral vaporesection of the prostate (ThuVARP) versus standard transurethral resection of the prostate (TURP) in men with benign prostatic obstruction (BPO). Randomisation is at the patient level so men were randomised 1:1 to receive either ThuVARP or TURP.

This study is powered to establish equivalence in clinical improvement. We are specifying differences in Qmax and IPSS of no greater than 4 ml/s and 2.5 units respectively, as demonstrating equivalence.

**Fig. 1:**
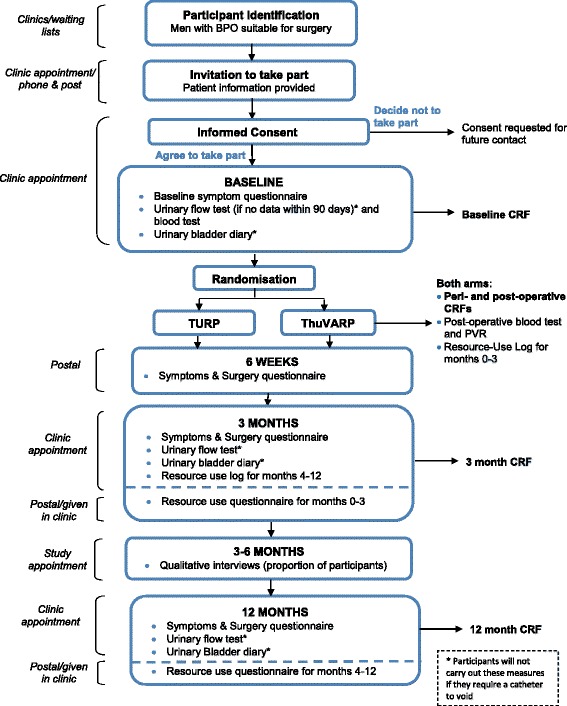
Study flow diagram. *BPO* benign prostatic obstruction, *CRF* case report form, *PVR* post-void residual, *ThuVARP* thulium laser transurethral vaporesection of the prostate, *TURP* transurethral resection of the prostate

Follow-up is at 6 weeks, 3 and 12 months after surgery for the patient-reported outcome (PRO) IPSS, and at 3 and 12 months for the maximum urine flow rate (Qmax), with 12 months being the primary endpoint. Patients are asked to complete other patient-reported outcomes at 6 weeks after surgery (by post), and after 3 and 12 months. The study flow diagram is provided in Fig. 1 and the Standard Protocol Items: Recommendations for Interventional Trials (SPIRIT) checklist in Additional file 1.

### Setting

Participants have been recruited and operated on at seven UK centres: four university teaching hospitals and three district general hospitals. Each of the centres in the trial performs between 150 and 400 benign prostate operations per year.

### Study duration

Recruitment for the trial began in June 2014 and completed at the end of December 2016. Twelve-month follow-up for all participants will complete by the end of November 2017.

### Participants

As this is a pragmatic trial, it includes men who are suitable for TURP referred to secondary care for assessment with a view to requiring BPO surgery, presenting with either bothersome LUTS or urinary retention secondary to BPO.

#### Inclusion criteria

- Men who were suitable for TURP, either in urinary retention or with bothersome LUTS, secondary to BPO.

#### Exclusion criteria

Patients with:

- Neurogenic LUTS
- Prostate cancer
- Previous prostate or urethral surgery
- A prostate-specific antigen (PSA) outside of the normal age-related range and who had not had prostate cancer excluded
- Men who were unable to give informed consent or complete trial documentation

### Interventions

Patients were randomised 1:1 to receive either the TURP or ThuVARP procedure. As this is a pragmatic study, centres continued to use their usual practices, for example, with respect to whether or not they did pressure-flow urodynamics as part of patient selection, or how they undertook the TURP procedure, e.g. use of monopolar or bipolar TURP. All trial surgeons underwent training on the ThuVARP technique as described below.

Patients undergoing concomitant procedures during their BPO surgery were included in the trial, and details of their additional surgery recorded.

All adverse events are recorded and serious adverse events notified to the appropriate authorities (Research Ethics Committee and Sponsor) within specified timelines.

### Surgeon training on the laser

ThuVARP uses laser technology to vaporise and resect the prostate while TURP uses electric current to resect the prostate. ThuVARP essentially uses the same surgical skill-set as for the TURP procedure which is part of core practice for all urologists, including trial surgeons who performed both procedures. The experience of the Chief Investigator and other urologists, in the UK and Europe, indicate that a maximum of 15 ThuVARP laser cases can assure competence in the ThuVARP laser procedure.

All surgeons were mentored by the Chief Investigator (CI) or another Principal Investigator (PI) already certified as competent with the ThuVARP technique, and certified by an independent assessor, using standard criteria, before the official study commenced. First, surgeons observed the CI/PI performing one to two cases. The CI/PI then observed the surgeons in each centre perform two to five cases during site visits. The surgeons then performed five to ten cases without supervision, following their respective Trust’s clinical governance and audit guidelines. Competency was assessed with the Intercollegiate Surgical Curriculum Programme work-based assessment (ISCP-WBA) by an independent assessor. If competency was not achieved at this stage, then further cases would have been observed and training provided by the CI until the competency criteria were met, however in practice all surgeons were signed off as competent at their first assessment.

### Withdrawal

Participants remain in the trial unless they choose to withdraw or if they are unable to continue for a clinical reason. If a participant withdraws consent, participant questionnaires are not collected. However, permission is sought for the research team to continue to collect outcome data from their health care records. Participants are informed in the Patient Information Sheet (PIS) that they have the right to withdraw all personal data held by the study, but otherwise this data is retained.

### Outcome measures

Two key co-primary outcomes were selected, measured at 12 months, based on a well-established and validated patient-reported outcome (PRO), the International Prostate Symptom Score (IPSS) [9], and the urodynamic clinical measure of maximum urine flow rate (Qmax: ml/s) which is used in all BPO trials. These outcomes address the primary research question for the trial.

The IPSS and Qmax are internationally accepted, and the most frequently used primary outcomes in BPO studies, thereby making results from this study comparable to others. There are no core outcomes measures for BPO listed in the COMET Initiative website.

Key secondary outcome measures include other well-validated PROs and answer the trial research questions:

1. Clavien-Dindo classification of surgical complications [10]
2. Length of hospital stay and transfusion rates
3. International Consultation on Incontinence Questionnaire – Male Lower Urinary Tract Symptoms (ICIQ-MLUTS) (for symptom bother)
4. International Index of Erectile Function (IIEF) and ICIQ-MLUTSsex (measures of erectile function)
5. ICIQ-LUTSqol (condition-specific quality of life score)
6. EuroQol Group’s five-dimension health status questionnaire (EQ-5D-5L) (preference-based general quality of life measure)
7. ICIQ-satisfaction (measures satisfaction with surgery outcomes) to assess the full impact of the intervention on patients and the NHS
8. Resource use for the year following randomisation to inform the cost-effectiveness analysis
9. Interviews following surgery, for qualitative analysis of men’s experiences of both procedures

In addition to these key secondary outcomes, other secondary outcomes are:

1. Post-operative catheterisation time
2. Haemoglobin (blood loss during surgery)
3. Serum sodium (absorption of irrigation fluid)
4. Post-void residual urine

### Assessment and follow-up

**Fig. 2:**
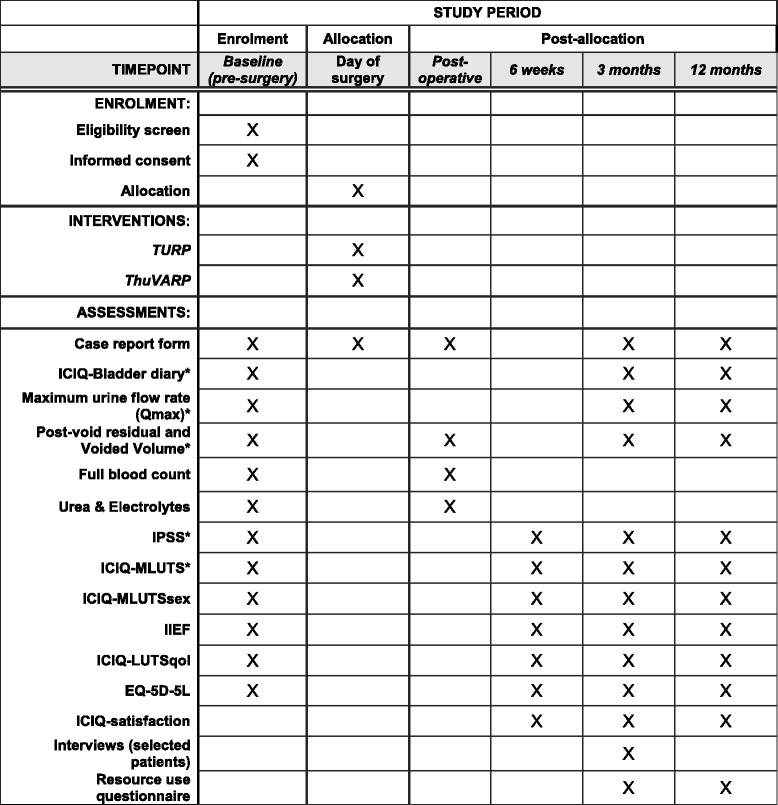
Schedule of enrolements, interventions and assessments

The timing of our outcome measurements are summarised in Fig. 2 (schedule of enrolment, interventions, and assessments) and described below.

#### Clinical outcomes

Urine flow rate (Qmax), post-void residual (PVR) and voided volume (VV) were measured before surgery in men who were able to void without a catheter. The most recent existing measures were used if they were performed within 90 days of informed consent. PVR and VV were measured post-operatively, and Qmax, PVR and VV at 3 and 12 months post-surgery. These follow-up flow measures are not collected if men are catheterised, but trial without catheter (TWOC) data is recorded.

Blood parameters were also measured at baseline and post-operatively, including full blood count (FBC) and urea and electrolytes (U&Es).

#### Patient reported outcomes

Participants receive the UNBLOCS Symptoms/Surgery Questionnaire at baseline in clinic, 6 weeks post-surgery (by post), and at 3 and 12 months post-surgery in clinic (with the option to take home and return in pre-paid envelope if preferred). The questionnaire contains questions from standardised outcome instruments for urinary and sexual symptoms (listed in Fig. 2) and the baseline questionnaire had versions for men who were using a catheter and those who were not, as some items were not relevant for catheterised patients. Participants who are catheterised at the time of the 3- or 12-month questionnaires are instructed to only answer the questions that they feel able to. The Bladder Diary was given to participants at their baseline clinic and at their 3 and 12 months clinics if they do not require a catheter to void at the time. The Bladder Diary is used to record fluid intake, frequency and volume of micturition and bladder sensation and use of pads over 3 days, and is returned in a pre-paid envelope.

#### Economic data collection

Details of initial hospital stay resource use, e.g. operation duration; operating staff; consumables; the time spent in recovery; length of stay on different wards; overall length of stay and treatment for complications, were collected on study-designed case report forms (CRFs) by the research nurse at the time of the inpatient/day-case stay for the initial surgery. Hospital patient-linked information costing systems are used if available to capture follow-up hospital use at the treating hospital. If this information cannot be accessed then subsequent inpatient stays, outpatient visits and procedures occurring at the treating hospitals are abstracted from the patients’ medical records and recorded on a CRF.

On discharge from hospital, and at 3 months follow-up the patients are given a study designed Resource Use Log (RUL) to be used as an aide memoire in which to record NHS and private community-based health care use, other NHS hospital health care use, medications, personal social service (PSS) resource use in addition to travel, time off work/usual activities and any other expenses resulting from their treatment [11]. These logs reflect the design of the 3-month and 12-month UNBLOCS Resource Use questionnaire. At 3 months and 12 months follow-up, participants are able to use the information from the RUL in order to complete the UNBLOCS Resource Use Questionnaire, which they take home from clinic, or receive by post along with a pre-paid return envelope. The EQ-5D-5L will be used to calculate quality-adjusted life years (QALYs). The new five-level EQ-5D is used in preference to the three-level one, owing to its improved discriminatory power [12].

#### Qualitative data collection

The main aims of the qualitative component, which is being investigated with in-depth semi-structured interviews with participants at between 3 and 6 months post-surgery are:

1. To explore patient experiences of ThuVARP and TURP
2. To explore determinants of patient satisfaction with the two procedures
3. To identify any differences in experience between men presenting with LUTS or urinary retention.

The basis of the interview schedule/topic guide is focused around these three themes and further informed by the literature and clinical experience of the co-applicants, but will also allow participants to address issues or concerns of particular relevance to their own experience. Interviews have been targeted at 3 to 6 months post-surgery to allow recovery from the operation and return to daily activities whilst also permitting good recall of the experience of the procedures and immediate sequelae.

Participants from both the ThuVARP and TURP intervention arms are recruited to take part in exploratory interviews. Study participants who provide consent to being approached for qualitative interviews are purposively selected to represent the two surgical interventions, two presentations for surgery (LUTS and urinary retention) and demographic characteristics such as age. This is included to better understand the differences between the two surgical procedures in terms of the individuals’ lived experience. In particular, the interview schedule focuses on the immediate experience surrounding surgery and features of the continued recovery and effects on daily life. This is important to capture contextual data to support interpretation and contextualisation of the trial quantitative outcomes.

Participants are also asked to articulate what their expectations of surgery were prior to the procedure and upon which factors they judged their perceived satisfaction, or dissatisfaction subsequent to the operation, and whether this was a dynamic or static decision that altered during the recovery period. This aspect of the investigation will focus on capturing both clinical and non-clinical determinants of satisfaction which will be linked to the quantitative analyses for interpreting the outcomes e.g. quality of life.

### Sample size

This study is powered to establish equivalence in clinical improvement. The Chinese trial [6] observed differences of 0.4 ml/s (95% CI: -2.0 to 2.8) in Qmax and 0.4 units (-0.7 to 1.5) in IPSS between ThuVARP and TURP. Variability (standard deviation; SD) in data at 12 months was approximately 6.0 ml/s (Qmax) and 3.0 units (IPSS), but previous trials of TURP report greater variability, around 9 ml/s (Qmax) and 5 units IPSS [13, 14].

We have specified differences of 4 ml/s in Qmax and 2.5 units in IPSS, as demonstrating equivalence. Equivalence studies often use an alternative hypothesis of a difference of zero between treatments. However, the Chinese trial observed differences of around 0.4 ml/s and 0.4 units for Qmax and IPSS. Incorporating these as alternative hypotheses ensures adequate power to demonstrate equivalence if treatments are indeed similar but not identical.

Assuming SDs of 9 ml/s for Qmax and 5 units for IPSS, the target sample size for patients needed to complete the 12-month follow-up was 163 patients in each group. Using NQuery Advisor, this will provide 85% power to demonstrate equivalence for Qmax and just over 90% power for IPSS, at a two-sided alpha of 5%. Assuming 20% loss to follow-up following randomisation, it was necessary to recruit 410 men in total. This loss to follow-up is a conservative estimate from our experience of previous trials. However, we are aiming to reduce loss to follow-up through letter, text and telephone reminders to patients.

### Randomisation

All men who entered the trial were logged with the central study office and given a unique, six-digit study (participant) identification number.

Participants were randomly allocated to treatment arms using an automated web/telephone randomisation system provided by the Bristol Randomised Trials Collaboration (BRTC). This took place in the anaesthetic room when the patient was anaesthetised.

Randomisation was stratified by centre and whether the patient was eligible due to bothersome LUTS or urinary retention, and random blocking was employed (of two, four and six randomly allocated).

### Blinding

To reduce bias in the assessment of outcomes, participants were not informed of their study arm allocation, although their general practitioner (GP) can access this information. Participants were informed that, although it would be preferred that they did not know which operation they have had; their GP will not be prevented from giving them this information if they request it. We anticipated that some men will ask for, or discover, their allocation at some point during the study and we will be asking them to reveal when and how they became aware of this in the 12-month follow-up questionnaire. Participants will be informed of the type of BPO surgery they received after receipt of their 12-month follow-up questionnaires and Bladder Diary, or 1 month after they have received a reminder for these.

### Statistical analysis

All data analysis will be in accordance with the Consolidated Standards of Reporting Trials (CONSORT) guidelines extension for non-inferiority and equivalence trials [15]. A full statistical analysis plan will be developed and agreed by the Data Monitoring Committee and the Trial Steering Group prior to undertaking any analyses of the trial data.

Descriptive statistics will be used to compare patient baseline characteristics between the two treatment groups. The primary comparative analyses will be conducted on an ‘as allocated’ basis and will employ multivariable linear regression to investigate equivalence in Qmax and IPSS between ThuVARP and TURP at 12 months. Analyses will adjust for stratification variables (centre and retention). Interpretation of results will focus on observed differences, and 95% confidence intervals for the between-group comparisons, to determine whether clinically important differences between ThuVARP and TURP are unlikely. Missing data will be imputed using multiple imputation modelling. Additional sensitivity analysis will explore the impact of missing data by using complete cases only. Sensitivity analyses will also consider adjustment for baseline measures of Qmax and IPSS (with clinically sensible values imputed for those with retention, for example a Qmax of zero) any variables demonstrating imbalance at baseline. Consideration will also be given to surgeon effects using mixed-effects models.

As randomisation will occur close to the time of surgery, a significant cross-over between treatment groups is thought unlikely, but any departures from protocol are likely to make the treatment groups more similar. In a superiority trial an ‘as allocated’ (or intention-to-treat) analysis is a conservative approach. In an equivalence trial, such as this, where the objective is to demonstrate that treatments have a similar effect, a per protocol analysis may be a more conservative approach, but prone to bias. An alternative is randomisation-based efficacy estimators (complier average causal effect models (CACE)) which maintain randomisation [16]. If protocol deviations occur we will conduct sensitivity analyses to assist with the interpretation of the primary result, these will include CACE and per protocol analyses with discussion of any likely bias in the resulting estimates.

Analyses of secondary analyses will employ linear, logistic or multinomial regression models as appropriate. Repeated measure models will explore any treatment-time interactions considering the 6 weeks and 3 months data in addition to baseline and 12 months. Potential effect modifiers, selected a priori and informed by previous evidence, will be explored using formal tests or interaction. These will include:

- Clinical diagnosis at baseline of LUTS secondary to BPO or urinary retention (stratification variable)
- Pre-operative prostate size measured by digital rectal examination
- Age
- Patient co-morbidities at baseline (Charlson Comorbidity Index)
- Conduct of TURP procedures; whether monopolar or bipolar TURP
- Length of stay of procedures; including whether day-case or inpatient

Interpretation will focus on the confidence intervals only and will be hypothesis-generating since the trial is not powered for such analyses. No interim analyses are planned.

### Economic data analysis

The trial includes a formal economic evaluation comparing the costs and cost-effectiveness of the interventions from an NHS and broader societal perspective (with personal social service and patient costs reported separately). Only resources used in relation to the treatment of LUTS, or urinary retention secondary to BPO, will be analysed from randomisation at the time of surgery, to 12 months follow-up. The cost of the interventions and the use of primary and secondary NHS services by the men, personal and social service costs, costs to the men arising from their treatment (e.g. travel, over-the-counter medication) and productivity costs will be estimated through the collection of resource-use data as outlined earlier and the valuation of these data.

Micro-costing of the initial hospital stay will be needed and therefore Trust finance departments of the participating hospitals will be approached in order to value the initial NHS resources used. All other resource use will be valued using information from the laser company, routine sources and information from the patients themselves.

The EQ-5D-5L is administered at baseline and at 6 weeks, 3 and 12 months after the operation. These values will be transformed into utility scores [17] and individual QALYs will be calculated using the area under the curve approach.

Initially, regression techniques adjusting for pre-specified baseline characteristics, randomisation variables and a centre effect will be used to evaluate the difference in costs. Boot-strapped confidence intervals will be used, given the potential non-normality of the cost data. The same model will be used to evaluate the difference in QALYs. The differences in terms of the two primary outcomes will be evaluated according to the statistical analysis plan.

For the base case economic analysis, for the two perspectives, cost-effectiveness will be assessed using the Net Benefit framework over a range of values for the QALY.

A secondary economic analysis, for the two perspectives, will be conducted in which the outcomes will be the co-primary outcomes of the trial (i.e. IPSS and urine flow, Qmax). For this analysis the differences in costs and effects will be examined. If one arm is dominant (i.e. less costly and more effective) no further incremental analysis will be conducted. Otherwise an incremental cost-effectiveness ratio (ICER) will be calculated. Seemingly unrelated regressions (SUR) will be used, if appropriate, to account for the potential correlation between costs and the IPSS score/Qmax values.

Uncertainty will be addressed using cost-effectiveness acceptability curves and sensitivity analyses. One aspect of uncertainty is likely to be that of missing data. As with the main analysis, a pre-specified analysis plan will be created in which the plausible assumptions about missing data will be created and tested using these assumptions within the sensitivity analyses.

Additionally the costs of surgeon training for the ThuVARP laser technique will be estimated. This will be reported to allow policy makers to more accurately estimate the costs of service reconfiguration if the ThuVARP laser is shown to be equivalent to the TURP. These costs will also be incorporated in one of the sensitivity analyses.

No modelling has been specified within this evaluation, as the work is seen as a definitive trial, and experience has shown that most uncertainty in relation to cost differences will be captured within the first 12 months, the duration of this trial.

### Qualitative data analysis

A standardised approach will be employed to explore the interview data in accordance with published qualitative research methods. Face-to-face patient interviews will be conducted where possible, with telephone interviews included for other study sites, which will be carried out by an experienced qualitative researcher. Interviews will be digitally recorded, transcribed verbatim and uploaded into a qualitative software package to aid data management (QSR NVivo 10). Analyses will be conducted by the qualitative researcher according to the principles of thematic content analysis. Recordings will be listened to and transcripts read and re-read for familiarisation. Segments of text will be ‘coded’ by assigning descriptive labels. Codes will be grouped on the basis of shared properties to create themes, and coded transcripts will then be examined and compared to inductively refine and delineate themes (constant comparison). A subset of interviews will be independently analysed by a second study researcher and coding discrepancies discussed to maximise rigour and reliability. Plausibility of data interpretation will be further discussed between the study team, including the clinical co-applicants, throughout the analyses. Descriptive summary accounts of the interviews will be prepared.

Theoretical purposive (non-probability) sampling will be used, where explanations, developing to describe the data during analyses, guide further sampling and data collection. Maximum variation sampling will also ensure the diverse characteristics of the population are sampled (e.g. participants varying in age, clinical history and surgery received). Sampling and analyses will continue in iterative cycles until no new themes are emerging and established themes cease evolving: data saturation. It is anticipated that approximately 30–40 participants will be required, with up to 20 per procedure to allow for sampling of those with LUTS and urinary retention as the reason for their treatment.

## Discussion

This article describes a multicentre randomised trial to compare ThuVARP to the current gold standard TURP surgery for benign prostatic obstruction. The aim is to determine the relative clinical and cost effectiveness of the two alternative procedures, within the NHS.

The most significant challenge for the trial, during the recruitment phase, has been the capacity of participating Trusts to undertake the required surgery. Randomisation occurs in the operating theatre whilst the patient is under anaesthetic. Operating theatre availability and hospital bed pressures within Trusts, particularly during the winter months, slowed recruitment for this elective procedure, despite a ready pool of willing participants. Protected research operating time secured at two participating Trusts, the advent of day case procedures and multiple surgeons at sites, helped to overcome these challenges, albeit with additional time for recruitment required. It should be noted that the very difficulties experienced in conducting this trial, with the shortage of hospital beds impacting on the capacity for Trusts to operate, are the very same issues which ThuVARP has the promise to address, with its potential for shorter hospital stays, and shift to day case procedures.

This randomised study will provide the high-quality evidence, in an NHS setting with a range of patient-reported, clinical and cost-effectiveness outcomes, which will underpin and inform future NICE guidance in a timely manner.

### Trial status

The UNBLOCS trial began recruitment in June 2014, with the first participant enrolled on 23 July 2014. Recruitment completed on 31 December 2016.

## Additional file

Additional file 1: SPIRIT checklist. (DOC 121 kb)

## Abbreviations

BPO: Benign prostatic obstruction
BRTC: Bristol Randomised Trials Collaboration
CI: Chief Investigator
CONSORT: Consolidated Standards of Reporting Trials
CRF: Case report form
DMEC: Data Monitoring and Ethics Committee
EAU: European Association of Urology
EQ-5D-5L: EuroQol Group’s five-dimension health status questionnaire
FBC: Full blood count
GP: General practitioner
HES: Hospital Episode Statistics
HoLEP: Holmium Laser Enucleation of the Prostate
HTA: Health Technology Assessment
ICIQ-LUTSqol: International Consultation on Incontinence Questionnaire – Lower Urinary Tract Symptoms Quality of Life
ICIQ-MLUTS: International Consultation on Incontinence Questionnaire – Male Lower Urinary Tract Symptoms
ICIQ-MLUTSsex: International Consultation on Incontinence Questionnaire – sexual function associated with Male Lower Urinary Tract Symptoms
ICIQ-satisfaction: International Consultation on Incontinence Questionnaire – Satisfaction
IIEF: International Index of Erectile Function
IPSS: International Prostate Symptom Score
ISCP-WBA: Intercollegiate Surgical Curriculum Programme work-based assessments
ISRCTN: International Standard Randomised Controlled Trial Number
LUTS: Lower Urinary Tract Symptoms
NHS: National Health Service
NICE: National Institute for Health and Care Excellence
NIHR: National Institute for Health Research
NRES: National Research Ethics Service
PI: Principal Investigator
PIS: Patient Information Sheet
PRO: Patient-reported outcome
PSA: Prostate-specific antigen
PSS: Personal Social Services
PVR: Post-void residual
QALY: Quality-adjusted life years
Qmax: Maximum urine flow rate
RCT: Randomised controlled trial
RUL: Resource Use Log
SD: Standard deviation
ThuVARP: Thulium laser transurethral vaporesection of the prostate
TURP: Transurethral resection of the prostate
U&Es: Urea and electrolytes
UK: United Kingdom
UNBLOCS: UriNary oBstruction relieved by Laser Or Conventional Surgery
VV: Voided volume
